# Recent Advancements in the Fabrication of Functional Nanoporous Materials and Their Biomedical Applications

**DOI:** 10.3390/ma15062111

**Published:** 2022-03-13

**Authors:** Matthew Hadden, David Martinez-Martin, Ken-Tye Yong, Yogambha Ramaswamy, Gurvinder Singh

**Affiliations:** 1 The School of Biomedical Engineering, The University of Sydney, Sydney, NSW 2006, Australia; matthew.hadden@sydney.edu.au (M.H.); david.martinezmartin@sydney.edu.au (D.M.-M.); ken.yong@sydney.edu.au (K.-T.Y.); 2 Sydney Nano Institute, The University of Sydney, Sydney, NSW 2006, Australia

**Keywords:** nanoporous materials, additive manufacturing, dealloying, nanoporosity, hierarchical nanoporous

## Abstract

Functional nanoporous materials are categorized as an important class of nanostructured materials because of their tunable porosity and pore geometry (size, shape, and distribution) and their unique chemical and physical properties as compared with other nanostructures and bulk counterparts. Progress in developing a broad spectrum of nanoporous materials has accelerated their use for extensive applications in catalysis, sensing, separation, and environmental, energy, and biomedical areas. The purpose of this review is to provide recent advances in synthesis strategies for designing ordered or hierarchical nanoporous materials of tunable porosity and complex architectures. Furthermore, we briefly highlight working principles, potential pitfalls, experimental challenges, and limitations associated with nanoporous material fabrication strategies. Finally, we give a forward look at how digitally controlled additive manufacturing may overcome existing obstacles to guide the design and development of next-generation nanoporous materials with predefined properties for industrial manufacturing and applications.

## 1. Introduction

Nanoporous materials are categorized as an important class of nanostructured material that possess unique surface and structural characteristics, including high surface area, tunable pore sizes, tunable pore geometries, as well as surface topographies with porous architectures. These unique characteristics of nanoporous materials underline their applications in various fields, such as ion-exchange [1], separation [2], catalysis [3], sensors [4], water purification [5], CO_2_ capture and storage [6], renewable energy [7,8], targeted drug delivery [9], tissue engineering [10], and implants [11]. For example, the presence of highly active low-coordinated atoms (i.e., atoms with lower numbers of bonds such as atoms on surfaces, steps, and kinks) on interconnected curved backbones of nanoporous structures make them suitable for catalytic applications. Their extensive porous networks facilitate the mass transfer of reactants from the exterior surfaces to the interior surfaces, thus, enhancing the catalytic reaction rate, even at low temperatures. The lightweight and excellent mechanical properties of nanoporous materials make them suitable for use in medical implants. Furthermore, the design of nanoporous materials with variations in pore size can reduce the response time and improve sensitivity of sensing and microfluidic devices. Nanoporous materials can be used as a platform to understand and study guest–host reactions [12,13], chemical reactivities in confined environments [14], and chemical reactions involved in the synthesis of nanomaterials (nanoparticles, nanowires, and quantum dots) [15].

In general, nanoporous materials can be defined as any material with a pore size of nanoscale dimension (100 nm or less) in their structures. However, these nanoporous materials can be developed and classified into categories based on pore size, structure of the pores, crystallinity of the materials, and type of materials. In the case of pore size, these materials are subdivided, based on the international union of pure and applied chemistry (IUPAC) nomenclature, into three different categories: microporous (pore size <2 nm), mesoporous (pore size ~2–50 nm), and macroporous (pore size >50 nm) [16]. The classification of porous structures is generally based on disordered and ordered systems. Compared with disordered structures, ordered nanoporous materials are desirable for optoelectronic applications as they provide periodicity of pore sizes and, thus, facilitate greater control over their interactions with electromagnetic waves [17]. Furthermore, nanoporous materials can be categorized based on the crystallinity (i.e., amorphous vs. crystalline) of the materials used. Amorphous nanoporous materials have a hierarchy of pore sizes (a wide distribution of pore sizes) and are suitable for heterogenous catalytic reactions. These amorphous nanoporous materials are comprised of cross-linked polymers, carbon, and porous aromatic frameworks. Crystalline nanoporous materials have a narrow pore size distribution and are suitable for molecular sieving filtration where uniformity in pore size is important [18,19]. Zeolites, metal organic frameworks (MOFs), and covalent organic frameworks are examples of crystalline nanoporous materials [20,21,22]. Nanoporous materials can also be subdivided based on the material type, i.e., inorganic (zeolites, mesoporous silica), metal-organic frameworks (MOFs), and covalent-organic frameworks (COFs). MOFs are considered to be a new class of crystalline nanoporous materials with rigid nanopore structures. MOF-based nanoporous materials are formed by the interconnections of inorganic constituents (metal nodes) with organic molecules. The tunable chemical functionalities with well-defined nanopore size and geometries makes them suitable for several practical applications, such as gas separation [23], gas capture [24], energy storage [25], and catalysis [26,27,28]. A recent review provided the updated progress in various strategies for developing MOF-based nanoporous materials [23,29].

Since the synthesis of mesoporous silica was first reported in the 1990s, significant progress has been made in developing strategies for fabricating nanoporous materials from a wide range of materials, including metal, metal oxides, and polymers [30,31]. The most common strategies adapted for fabricating such materials include dealloying, soft and hard template methods, physical vapor deposition, nonsacrificial templating, and block copolymers and colloidal self-assembly. These strategies have been used to fabricate ordered, disordered, and hierarchical nanoporous materials with tunable pore sizes, shapes, and relative orientation of pores at different length scales. In recent years, several reviews published on nanoporous materials have primarily focused on selective modification of nanoporous materials, synthesis, and characterization of specific types of nanoporous materials and their applications [8,17,31,32,33,34,35,36,37,38,39,40]. Indeed, the field of nanoporous materials is progressing at a rapid pace with more emphasis on developing new fabrication strategies. These strategies must be scalable with a viable cost to achieve long-term commercial impact on broader applications. This review aims to provide a comprehensive analysis of recent developments in techniques adapted to fabricate/synthesize nanoporous structures from a wide spectrum of materials. The review summarizes recent progress on the synthesis of nanoporous materials by various methods, including dealloying, templating, microwave-based, and ion beam- and laser-induced fabrication technologies (see Table 1). It provides insights and highlights the perspective and future research direction of nanoporous material developments and fabrication technologies.

## 2. Dealloying for Fabricating Nanoporous Materials

Dealloying is considered to be a versatile and robust top-down approach to fabricate disordered and hierarchical nanoporous materials with tunable pore sizes with few nanometers. In this process, the selective dissolution and/or leaching of less noble metals from an alloy or composite material induces three-dimensional (3D) porosity in materials. This was first demonstrated in the Au-Ag alloy system. The selective dissolution of the less noble metal (Ag) due to acid treatment resulted in the formation of 3D nanoporous metals [41]. The technique can be applied to fabricate nanoporous materials with macro-scale dimensions and these materials can be engineered to different shapes prior to dealloying. The precursor alloys should be homogenous to fabricate nanoporous materials with desirable structural properties and minimal mechanical damage. Various dealloying approaches, such as chemical, electrochemical, liquid metal, and vapor phase dealloying have been established to create nanoporosity in both metals and metal oxides. 

### 2.1. Chemical Dealloying

**Figure 1:**
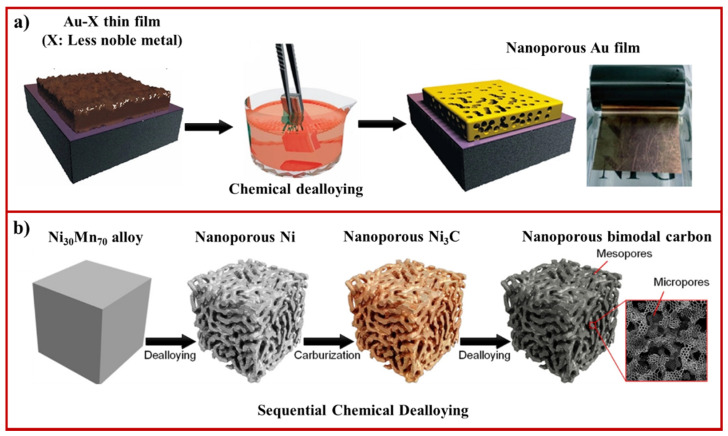
(**a**) Schematic illustration showing the fabrication of nanoporous Au thin film by selective chemical dealloying of Au-Ag film in acidic medium (adapted from [56], Copyright 2015, American Chemical Society); (**b**) schematic depicting the fabrication of 3D bimodal nanoporous amorphous carbon by sequential chemical dealloying (adapted from [57], Copyright 2021, American Chemical Society).

Chemical dealloying has been widely used to fabricate nanoporous metals and metal oxides by selective etching/leaching of less reactive species in an acidic or basic medium (Figure 1a) [42,43,44]. This process has been applied to fabricate homogenous and crack-free nanoporous Au from different precursor alloys, including Au-Ag [45], Au-Ni [46], and Au-Cu [47]. Nanoporosity has evolved as a result of the dissolution of the less noble metal (Ag) layer-by-layer, followed by surface diffusion and reorganization of more noble metal (Au) at the interface [48]. Therefore, the nanoporosity in materials can be associated with the surface mobility (diffusivity) of the more noble metal. However, the surface mobility of metal atoms should be optimal because a high surface diffusion rate of metal atoms can lead to coarsening of the nanoporous structure, which can cause undesirable effects, such as reducing the surface energy and/or area. This can cause the degradation of physical properties over time, even at room temperature. The problem of coarsening can be overcome by slowing the surface diffusion, performing dealloying at low temperatures, and the addition of a third noble metal (Pt) component to precursor alloy (Au-Pt-Ag) [49,50]. The approach has been extended to design nanoporous Pt, Ag, Pd, and Cu through chemical dealloying of Al-based precursor alloys [42,51,52], as well as bimetallic (CuTi, PtTi) [53,54] and trimetallic (Al_2_CuTi) nanoporous materials with pore sizes in the range of 10–20 nm [55]. In recent work, Hyun et al. fabricated trimetallic nanoporous materials from the dealloying of Al-Cu-Ti-based precursor alloy [55]. They showed that adding minor element (4–6% of Ni and Ag) precursor alloy significantly suppressed dealloying kinetics (lowering the diffusion rate of atoms), thus, reducing the coarsening effect. Chemical dealloying has also been employed to synthesize nanoporous metal oxides by exposing composite materials consisting of more reactive alkaline-earth metal oxides with less reactive transition metal oxides to an acidic environment [32,44].

In addition to the fabrication of nanoporous materials, the top-down chemical dealloying approach can also be employed to tune surface structures of nanoporous metals. When the dealloying process is carried out in the presence of pyrogallol and sodium citrate surfactants in an acidic environment, it results in the formation of nanoporous materials enriched with {111} and {100} structures, respectively [58]. The preferential and strong binding of pyrogallol molecules and citrate ions to the {111} and {100} are expected to modify the surface energy by stabilization of {111} and {100} facets and, thus, alter the surface diffusion and rearrangements of the more noble metal. To date, chemical dealloying has been used to fabricate nanoporous materials from a range of metallic elements in the periodic table. However, fabricating non-metallic nanoporous materials poses significant challenges due to high chemical stability and low diffusivity. Recently, Lan et al. used cobalt (Co)-boron (B) precursor consisting of Co/B at a ratio of 35:65 formed by the rapid solidification and its dealloying in 1 M HCl solution at 60 °C for 12 h, which led to the formation of 3D nanoporous boron via selective leaching of Co [59]. The pore size and porosity of nanoporous boron could be tuned by controlling the volume fraction of the CoB phase. In another recent work, Han et al. adapted a sequential chemical dealloying approach and demonstrated the production of 3D bimodal nanoporous amorphous carbon (Figure 1b) [57]. The process involved the formation of nanoporous Ni by selective removal of Mn from Ni-Mn alloys, followed by the conversion of nanoporous Ni (mesopores of about tens of nanometer) into metastable Ni_3_C bicontinuous nanoporous materials. Further chemical dealloying of Ni_3_C led to the formation of bimodal nanoporous amorphous caron (second level micropores of size smaller than 1 nanometer). The chemical dealloying technique has also be used for fabricating nanoporous glass materials. For example, Jiao et al. demonstrated the fabrication of tunable nanoporous metallic glasses by selective dissolution for ultrafast hydrogen uptake as compared with nonporous glassy alloy [60]. Recently, Rysiakiewicz-Pasek et al. showed the synthesis of porous glass-based nanocomposites by the selective dissolution of iron from silica-iron composites in a basic environment (potassium hydroxide solution) for sensing applications [61,62].

### 2.2. Electrochemical Dealloying

Electrochemical dealloying is a process in which the selective dissolution of a more electrochemically active metal of low standard electrode potential from a homogenous alloy leads to the formation of 3D nanoporous structures. In this process, the elements involved in the precursor alloy should have different chemical reduction potentials. For example, nanoporous Ni can be fabricated by electrochemical etching of Cu from the homogenous Ni_x_Cu_1-x_ alloys because Cu has a lower standard electrode potential than Ni [63]. The technique has been employed to produce nanoporous Au [64], Ag [65], Pt [66], Pd [67], and Cu [68], as well as bimetallic nanoporous MnFe from Mn-Fe-Cu alloy [69] and PdAu [70]. Erlebacher et al. established a model to explain the nanoporosity evolution during electrochemical dealloying [41]. According to this model, when the highly active metal dissolved from the surface of the precursor alloy, atoms of lower active metal diffused along the solid–liquid interface, coalescing into clusters and, thus, exposed areas of more noble metal to the electrolyte solution. Therefore, competition between the rate of dissolution and interface diffusion mechanism resulted in the formation of a stable nanoporous structure. For both electrochemical and chemical dealloying techniques, the uniformity and size of the pore are influenced by the phase homogeneity. The pore size and hierarchy of the porous structures can be tuned by varying the phase ratio and etching conditions. The techniques are both time consuming and endures a limitation from recovery of sacrificial phase, industrial scalability, and the use of harsh chemicals.

Electrochemical dealloying offers several advantages over chemical dealloying, such as better control over porosity and chemical composition by adjusting the electrolyte composition, time, and dealloying potential (i.e., the formation of much finer nanoporous structure at higher applied potential due to faster rate of dissolution and diffusion). In recent years, electrochemical dealloying processes have been modified to produce nanoporous materials from air- and water-sensitive metals, which holds great potential towards producing hydrogen on-demand by hydrolysis and energy storage applications. Fu et al. synthesized 3D bicontinuous nanoporous Mg (pore sizes ~20–30 nm) from Mg-Li parent alloy using air-free electrochemical dealloying [71]. In this process, the sacrificial component of the parent alloy (Li) was recovered by using a pure Li foil as a counter electrode, thus, making the process eco-friendly. A similar method has also been used to fabricate oxide-free nanoporous Sn [72], Li [73], Si [74], and Al [75] in anhydrous electrolytes under an inert atmosphere.

### 2.3. Liquid Metal Dealloying

**Figure 2:**
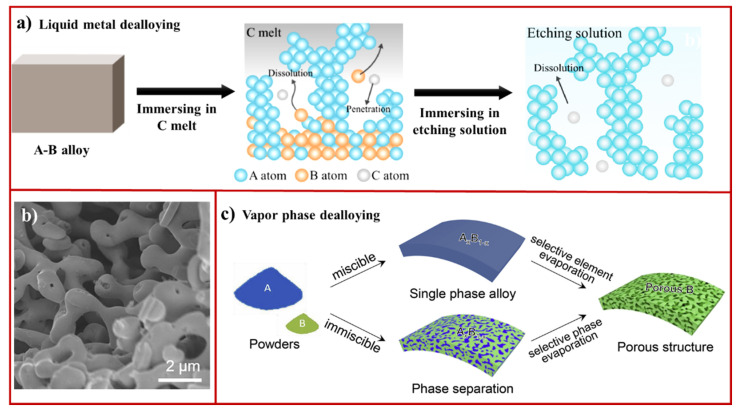
(**a**) Schematic illustration showing liquid metal dealloying working principle (adapted from [38], Copyright 2021, Elsevier); (**b**) scanning electron microscopy (SEM) image of nanoporous TaTi after the removal of Cu from the TiTa-Cu composite (adapted from [76], Copyright 2020, Elsevier); (**c**) scheme displays the principle of vapor phase dealloying (adapted from [77], Copyright 2019, Elsevier).

Liquid metal dealloying is a promising technique that has been adapted for fabricating nanoporous materials which uses metallic melts as the dealloying medium instead of acidic and basic media [38]. This technique relies on the solubilities of each element of precursor alloy into a liquid metal medium. The basic principle for liquid metal dealloying is illustrated in Figure 2a. As the prepared precursor alloy (A-B) is immersed in liquid C, element B is selectively dissolved into liquid C from the precursor alloy due to its solubility into liquid C and, thus, results in the formation of a 3D nanoporous structure. After completion of the dealloying process, the etching process is employed to recover the dissolved C and C-B phases from the solution. This method is more efficient and environment friendly because it allows the recycling of scraps.

Three important criteria must be taken into consideration for this technique. First, a dealloying reaction should be performed at a temperature where the medium becomes liquid, but the parent alloy remains solid. Therefore, the selection of an appropriate temperature is important. Second, the elements present in the precursor alloy should have a large solubility difference at dealloying temperature because the solubility of elements in dealloyed media can also be affected by the presence of other elements in the parent alloy. The third criterion is the selection of dealloying medium for recovering scrap elements. Since the discovery of liquid metal dealloying by Harrison and Wagner in 1959 [78] and, later, Wada et al. in 2011 [79], this technique has been employed to fabricate nanoporous structures from materials that cannot be dealloyed by chemical and electrochemical methods. For example, chemical and electrochemical methods cannot be used to fabricate nanoporous Ta and Ti due to oxidation and relatively low surface mobilities. In the last decade, this approach has been employed to fabricate nanoporous materials, including Ti [80], Ti alloys (TiZr, TiNb, TiFe, and TiTa) [76,81,82,83], FeCo [84], and Nb (Figure 2b) [85]. In addition to metals, 3D bicontinuous meso-macro porous graphite can be fabricated by dealloying of the Mn_85_C_15_ precursor alloy in metallic (Bi) melts at 1073 K [86]. An increase in temperature (>2273 K) led to more ordered 3D bicontinuous graphite formation. The rate of liquid metal dealloying is much faster than chemical dealloying because of its operation at elevated temperatures. However, the drawbacks of this method are unavoidable structural coarsening due to its operation at high temperatures, which can significantly influence the functional properties of nanoporous materials by reducing the surface area. To overcome this problem of thermal coarsening, a new material design strategy based on the use of high-entropy alloy has been investigated [87,88]. This has enabled the production of nanoporous materials with pore size of 10 nm at 837 K, thus, creating new opportunities for designing ultrastable nanoporous materials.

### 2.4. Vapor Phase Dealloying

Vapor phase dealloying utilizes differences in the vapor pressure of elements present in the precursor alloy to selectively evaporate and/or dealloy one component. In this technique, the precursor alloy is prepared by mechanically mixing the elements, followed by melt spinning to engineer a thin film (Figure 2c). Vacuum heating introduces nanoporosity in the materials by the selective evaporation of an element from the precursor alloy. Although the formation of porosity under vacuum was observed by Balluffi and Alexander in the 1950s [89], this technique did not received a great deal of attention until it was used to generate 3D bicontinuous nanoporous Co by high vacuum heating of the mechanically prepared precursor Co_5_Zn_21_ alloy [90]. Co and Zn had a wide difference in saturated vapor pressure over a range of temperatures, suggesting that the selective removal of Zn resulted in the evolution of nanoporous Co. The pore size can be controlled via vacuum pressure, temperature, and heating time. This approach was also expanded to fabricate nanoporous Ni, Ge, and Al from Ni-Zn, Ge-Zn, and Al-Zn, respectively [77,91,92]. This technique offers several benefits, including easy and cost-effective recovery of evaporated elements and the capability to fabricate nanoporous materials from less noble metals and low melting point elements independent of their electrical conductivity, electrochemical activity, and alloying miscibility. To date, the technique has been limited to only Zn-based alloy systems, and the versatility of this approach is yet to be demonstrated. The requirement of a vacuum chamber can also limit scalability and high-throughput production of nanoporous materials.

## 3. Templating

Templating is considered to be a promising approach for fabricating ordered, disordered, and hierarchical nanoporous metals and metal oxides with tunable pore sizes and pore geometries from a wide range of materials. A templating method involves three processing steps: (i) designing a soft or hard template, (ii) material deposition or chemical reduction to the template, and (iii) nanoporous structure formation after the removal of the template. The desirable materials can be deposited on the template via numerous strategies, including physical vapor deposition, electrochemical/chemical plating, sputtering, sol-gel method, or electron evaporation. The templating approach must also meet three essential criteria: (i) the infiltration of materials into the template to generate a continuous nanoporous structure; (ii) the stability of the template after deposition of the desired materials, and (iii) an appropriate template removal strategy to obtain stable nanoporous structures. In this section, we review various templating approaches utilized for synthesizing nanoporous materials.

### 3.1. Soft Template Method

**Figure 3:**
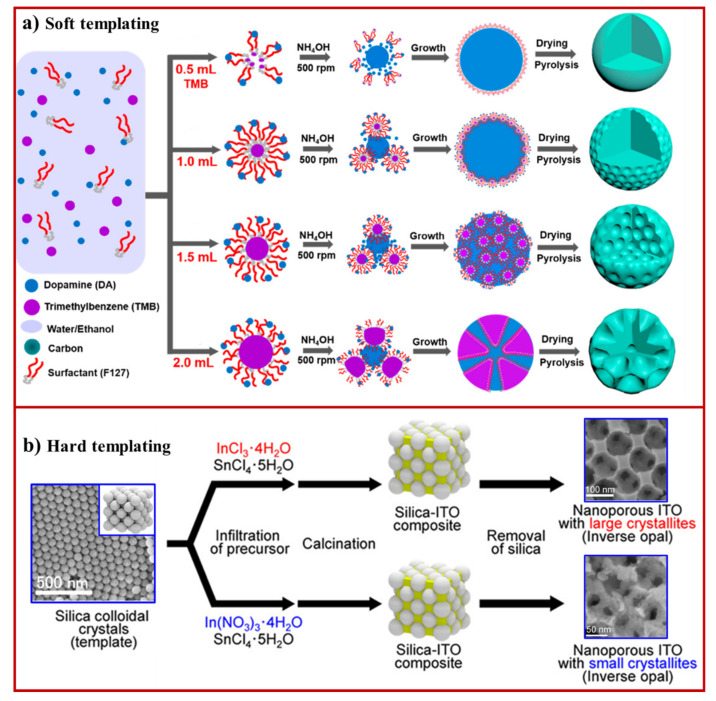
(**a**) Schematic illustration displaying the formation of nanoporous carbon in different morphologies depending on the ratio of surfactant to trimethylbenzene (TMB) in the soft micellar structure (adapted from [93], Copyright 2019, American Chemical Society); (**b**) fabrication of ordered nanoporous materials ITO using silica colloidal crystals as a hard template (adapted from [94], Copyright 2015, American Chemical Society).

The soft template method utilizes a micellar structure made from surfactants, and organic and block copolymeric molecules as a sacrificial template to fabricate nanoporous metals, metal oxides, and carbons. When these templates are exposed to precursor solution, precursor constituents interact with the template by weak noncovalent bonds, such as van der Waals interactions, hydrogen bonding, and electrostatic interactions. This approach offers great control over the geometry, pore size, and architecture of the nanoporous materials. Surfactants are amphiphilic molecules consisting of both hydrophobic and hydrophilic constituents. When these surfactants are dissolved in the solvent, these surfactant molecules are self-assembled into micellar structures with surface hydrophilic or hydrophobic groups depending on the polar or nonpolar solvent. The choice of appropriate surfactant is an essential step in designing nanoporous materials with desirable pore sizes and geometries. The soft templating approach has widely been explored to fabricate nanoporous carbon materials into different shapes by using surfactants, such as cetrimonium bromide (CTAB). The micellar templates of different morphologies such as lamella, disordered mesophases, and hexagonal have been fabricated by tuning the ratio of phenol/CTAB from 1:1 to 6:1 [35,93]. The thermal collapse of the micellar template resulted in the fabrication of nanoporous carbon. The use of block copolymers as soft templates has received significant attention in the research community because of their rich phase characteristics and tunable properties, facilitating their self-assembly into a range of macromolecular architectures [95,96,97,98]. For example, Peng et al. has demonstrated the synthesis of nanoporous carbon (pore size ~37 nm) in smooth, golf ball, multibranched, and dendritic morphologies by utilizing commercial triblock copolymer EO106-PO70-EO106 (F127) [95]. The size of morphologies was varied by adjusting the ratio of F127 to 1,3,5-trimethylbenzene solvent (Figure 3a). Similar F127 molecules were used as a soft template for the fabrication of mesoporous carbon in a single-step pyrolysis strategy [96]. The soft template approach is more suitable for synthesizing nanoporous carbon than hard template carbon because it eliminates the need for soft template removal after carbonization.

Soft template methods have also been employed to fabricate nanoporous metals and metal oxides. Krishnan et al. developed a cost-effective strategy to fabricate a polystyrene soft template of controlled porosity and pore size by solvent annealing, and then used it as a template to synthesize nanoporous SiO_2_ (sol-gel method), TiO_2_ (sol-gel method), and Ni (electroless plating) [99]. Control over pore size (from 10 nm to 35 nm) was obtained by varying the solvent annealing time from 5 min to 60 min. To produce hierarchical or bimodal nanoporous materials, the combination of two surfactant molecules of different molecular weights were used to design soft template. Recently, Fei et al. developed a single-step synthesis of hierarchical bimodal nanoporous carbon [100]. In this approach, the cooperative assembly of block copolymers of large and low molecular weight phenol-formaldehyde resin led to planet satellite morphologies, followed by carbonization leading to hierarchical nanoporous carbon. Microemulsion based soft templating has also been explored to fabricate nanoporous structures from polymers, inorganic and hybrid inorganic-organic composites [36]. In this process, the porous polymeric structures formed by polymerization of oil-in-water-in-oil emulsions were used to fabricate hierarchical nanoporous metal oxides, such as TiO_2_, Al_2_O_3_, ZrO_2_, and SiO_2_ [36]. Although the soft template-based approach is simple, the stability of soft templating has restricted its use in fabricating nanoporous materials.

### 3.2. Hard Template Method

The hard template (nanocasting) method of fabrication involves the use of relatively rigid structures to fabricate uniform and regular nanoporous materials. Examples of hard templates include porous silica [101], zinc oxide [102], alumina [103], zeolite [20] or self-assembled colloidal crystals [94]. The hard template method provides additional support to nanoporous structures to retain stable pore geometry even after high temperature or solution treatment of the template. Significant progress has been made to develop scalable strategies based on evaporation for constructing 2D and 3D self-assembled colloidal crystals [104,105]. These colloidal crystals can be used to fabricate ordered nanoporous materials with a long-range arrangement of pores within the structure. The pore size can also be adjusted by varying the size of particles used for designing self-assembled crystals. A range of techniques, including sputtering, electrochemical deposition, chemical vapor deposition, atomic layer deposition, and liquid precursor followed by calcination, are commonly used to infiltrate materials into the empty pores within colloidal crystals. This approach has successfully been applied to prepare metal and metal oxide-based nanoporous materials. Recently, Saito et al. fabricated highly ordered and crystalline nanoporous indium tin oxide (ITO) by using silica particle-based 3D colloidal crystal template [94]. 3D Nanoporous ITO with large and small crystallites were prepared by infiltration of indium chloride/tin chloride and indium nitrate/tin chloride precursors, respectively. After calcination of the infiltrated template, the template was removed using 2 M NaOH solution (Figure 3b). Interestingly, this study showed that thermal and electrical conductivity could be independently controlled by tuning the porous structures of ITO. Silica-based colloidal crystal template has also been used to fabricate novel organosilicon-based nanoporous materials formed by infiltration of precursors. One example is the crosslinking of poly[(mercaptopropyl)methylsiloxane] and 2,4,6,8-tetramethyl-2,4,6,8-tetravinylcyclotetrasiloxane by thiol–ene reaction [106]. For the colloidal crystal template made from polystyrene spheres, the template can be carried out by pyrolysis at 300 °C under an inert environment [17].

Among various hard templates, mesoporous silica (SBA-15), montmorillonite (MMT), porous anodic aluminum oxide (AAO), and magnesium oxide (MgO) have been widely used to prepare nanoporous materials. For example, Ling et al. used SBA-15 and MMT as hard templates to fabricate nanoporous carbon with a pore size distribution of 3.5 and 4.5 nm, respectively [101]. In this approach, vitamin B12 was used as a carbon precursor. When porous aluminum oxide (AAO) was used as a template, nanoporous carbon with pore sizes in the range of 50–200 nm was fabricated [37]. The results from these studies suggest that pore size and structural properties of nanoporous carbon depend on the properties of hard template type. Fan et al. employed a MgO hard template to fabricate a number of different types of nanoporous carbon [107]. These templates have also been used for composite/hybrid nanoporous materials. For example, SBA-15 templates have been used to fabricate CuMnO by infiltration of copper nitrate and manganese nitrate precursors and calcination at 450 °C for 5 h [108]. The resultant materials were washed with 2 M NaOH solution to remove the template. In a similar study, SBA-15 has been demonstrated to prepare ordered nanoporous cerium-rich copper oxide by infiltration of cerium and copper nitrate followed by calcination and NaOH-mediated template removal [109]. Other nonconventional hard templates, such as mesoporous carbon materials, have been used to fabricate nanoporous NiCoO materials [110].

## 4. Microwave-Based Fabrication of Nanoporous Materials

Microwave-based techniques has been used to fabricate nanoporous materials from various materials, such as polymers, metal oxides, silica, zeolites, and metal-organic frameworks. When microwave irradiation is exposed to a precursor material, the temperature increases upon microwave penetration (conversion of microwave energy to heat). The origin of microwave heating is caused by two mechanisms: ionic conduction (oscillation of cations or anions back and forth) and dipolar polarization (fluctuations/rotations of dipoles) [34]. Microwave heating increases the temperature of the reaction mixture (not vessel) uniformly as compared with conventional methods of heating, which are slow and nonuniform. In addition, the microwave approach can fabricate nanoporous materials with high yield, purity, and selectivity. Low energy microwave heating has been employed to synthesize highly crystalline mesoporous silica [111]. In the synthesis, microwave irradiation of 120 W was applied to precursors consisting of CTAB and sodium silicate, which led to the fabrication of highly ordered mesoporous silica of uniform pore size (~4 nm) in 30 min. The wall thickness of silica could be tuned from 0.34–0.36 nm to 0.78–0.90 nm by changing the microwave power, 120 W and 80 W, respectively (thinner nanoporous backbone at high microwave power). Attempts have also been made to fabricate heteroatom-doped mesoporous silica structures, thus, offering an avenue to alter their physical properties [112,113,114]. For example, Pd-doped mesoporous silica was prepared by adding porous silica in different weight ratios to the aqueous solution of palladium (II) chloride, followed by drying and calcination [112]. Such metal-doped mesoporous silica showed an enhanced hydrogen storage capacity of 1.74 wt.% at 25 °C as compared with 0.83 wt.% hydrogen storage capacity for pure silica under similar measurement conditions. 

In addition to generating nanoporosity in materials, microwave-based techniques can be used to develop functional nanoporous materials. For example, Huang et al. reported microwave irradiation to a sol solution prepared by mixing of Pluronic 123 with polyamine tetraethylenepentamine (TEPA), ethyl ether, and tetraethyl orthosilicate (TEOS), resulting in the formation of polyamine-modified mesoporous silica [115]. FTIR and NMR analyses confirmed the incorporation of polyamine to the pore channels of silica. Firstly, Pluronic 123 molecules bound to the surface silanol group present in the pores of silica by hydrogen bonding. The addition of polyamine invoked strong binding of polyamine to the surface silanol groups because silanol groups interacted more strongly with polyamine molecules than the Pluronic 123 molecules. The result from the findings revealed that the presence of Pluronic 123 molecules was essential to obtain efficient immobilization of amine groups to the silica surface. A microwave technique has been demonstrated to fabricate nanoporous carbon by carbonization and activation of lignin derived from waste pulping black liquor in a very short time (10–30 min) upon microwave irradiation via utilizing high content of inorganic salts [116]. Here, inorganic salts acted as microwave absorber. The technique has also been expanded to fabricate nanoporous aluminum oxide and titanium dioxide (TiO_2_) [117,118]. The exposure of microwave irradiation to the precursor solution prepared by mixing of titanium tetraisopropoxide with HCl or HBr resulted in the fabrication of nanoporous TiO_2_ with an average pore size of 14.5 nm [118].

## 5. Additive Manufacturing of Nanoporous Materials

Additive manufacturing involves a layer-by-layer deposition of material to design mechanically robust 3D physical objects from digital information. In recent years, additive manufacturing has revolutionized the design 3D materials of complex architectures and customized shapes because of low production cost. Three-dimensional printing technology has been used during the recent COVID-19 pandemic to meet the demands of the healthcare industry, from personal protection equipment to medical and testing devices, personal accessories, and isolation wards [119]. Progress has also been made to use additive manufacturing in combination with traditional nanoporous fabrication methods, such as dealloying, to produce a broad spectrum of ordered or hierarchical nanoporous materials with uniform porosity. Additive manufacturing offers several benefits, including low fabrication of nanoporous materials with tunable architecture, ability to produce materials over multiple lengths from meso- to macroscale, high level of reproducibility, industrial scalability, and mechanical robustness. The 3D printing approach also has the capability to fabricate compositional gradient hierarchical porous materials by changing the composition of relative ink during the printing process, thus, providing additional structural control with new functionality.

**Figure 4:**
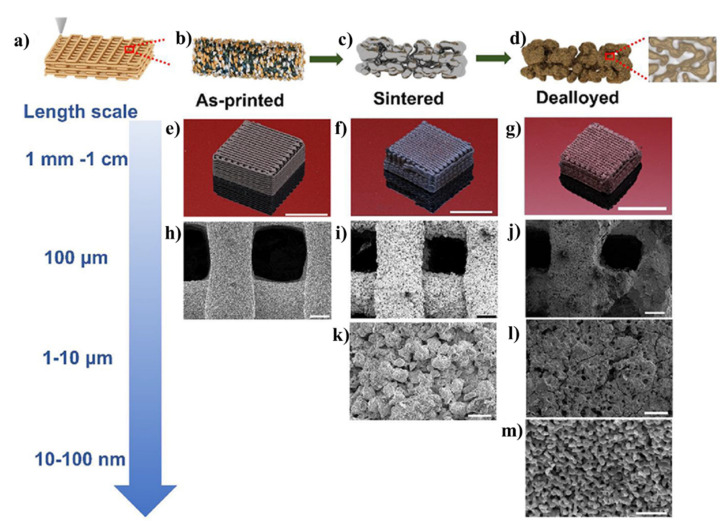
The fabrication of nanoporous materials of tunable porosity by 3D printing and chemical dealloying: (**a**,**b**) Digitally controlled macroporous 3D structures made from mixed Cu and Mn powder and polymer binder by direct ink writing; (**c**) thermal sintering at high temperature (1293 K) for 15 h to remove polymers and form Cu-Mn alloy; (**d**) nanoporous Cu fabrication by chemical dealloying in acidic environment to selectively remove Mn; (**e**–**g**) optical images of as-printed sintered and dealloyed sample. The scale bar in images (**e**–**g**): 10 mm. SEM images showing pore evolution after (**h**) 3D printing; (**I**,**k**) thermal sintering; and ( **j**–**m**) dealloying. The scale bar in (**h**–**j**) and (**k**–**l**) are 100 µm and 20 µm. The scale bar in (m) is 400 nm. Adapted from [120], Copyright 2020, Elsevier.

Mooraj et al. fabricated 3D hierarchical nanoporous metals with tunable porosity by combining 3D printing technology with chemical dealloying (Figure 4) [120]. The process involved direct ink writing 3D printing of Cu and Mn materials into a 3D object followed by thermal sintering to form 3D Cu-Mn alloy. Chemical dealloying of the 3D-printed Cu-Mn object in an acidic environment (hydrochloric acid) resulted in the formation of nanoporous Cu with pore sizes of 10–100 nm. This direct ink writing 3D printing technique combined with chemical dealloying has also been expanded to digitally controlled fabrication of nanoporous Au and AuAg in different geometries [121]. The results from this study have shown excellent long-term stability of nanoporous metals for catalytic applications as compared with Au nanoparticle-based catalysts. Recently, Cai et al. used a laser powder bed fusion (LPBF)-based 3D printing technique combined with chemical dealloying to produce hierarchical nanoporous Cu encapsulated with microporous diamond cellular structure-based catalyst for wastewater purification [122]. It has also been shown that the selective laser melting technique can be used to develop 3D-printed CuMn alloy and, thus, fabricate hierarchical nanoporous Cu with a pore size of 140 nm, after the selective dissolution of Mn from CuMn by chemical dealloying [123]. The results from this finding significantly showed improvement in the mass transport and mass-specific catalytic activity. In addition to exploring 3D printing for fabricating nanoporous metals, it has also been used to fabricate 3D nanoporous polymeric materials with complex geometries and controllable pore sizes from 10 nm to 1 µm via digital light processing and polymerization-induced phase separation [124]. Such hierarchical nanoporous polymers have demonstrated improved adsorption characteristics and cellular activity due to better pore accessibility and surface porosity. Through this technique, mechanically stable hierarchical nanoporous 3D objects were developed across multiple length scales ranging from micron to centimeter and applicable for a variety of applications, such as adsorption, filtration, drug delivery, and tissue engineering. Despite the advantages of controllable pore sizes, dimensions, and complex architectures, the technique has many limitations, foremost, the trade-off between mechanical stability of 3D-printed structures, and porosity/the distribution of pore sizes.

## 6. Ion Beam-Induced Fabrication of Nanoporous Materials

Progress has been made to fabricate nanoporous materials by ion beam irradiation on bulk materials or 3D self-assembled crystals made from nanoparticles. The ion beam technique is a top-down destructive technique involving the irradiation of high-energy ion beams, such as Ga^+^, He^+^, O^+^, Ar^+^, or Xe^+^. The proposed mechanism involved in the formation of nanopores using this method can be explained based on the combined effect of milling and sintering while being exposed to high-energy ion beam. The ion beam technique offers several advantages as compared with other fabrication strategies adapted for nanoporous materials, such as fine control over pore size distribution and an interconnected pore network by ion beam acceleration voltage, independent of material choices; no use of toxic chemicals; and no need for additional steps, such as chemical dealloying. Since the technique operates in a vacuum, air and water sensitive materials can also be used to fabricate nanoporous materials. Ion beam exposure to the materials may contaminate the backbone of nanoporous structures. However, the use of inert gas-based ion beams or thermal annealing can be used to remove ion contamination. Bischoff et al. used focused ion beam (Bi^+^) to fabricate amorphous porous germanium (Ge) [125]. They showed that the porosity and porous structure could be varied by changing the accelerating voltage of ion beams (30 kV and 60 kV) and the angle of incidence. In another work, the fabrication of nanoporous indium antimonide (InSb) was demonstrated by using focused ion (Ga^+^) beam [126]. The results showed a linear increase in the pore size by increasing the ion dose. However, nonporous InSb surface with pillar structures formed at a high ion dose instead of the porous structure. Therefore, the ion dose plays a vital role in controlling the porosity. 

**Figure 5:**
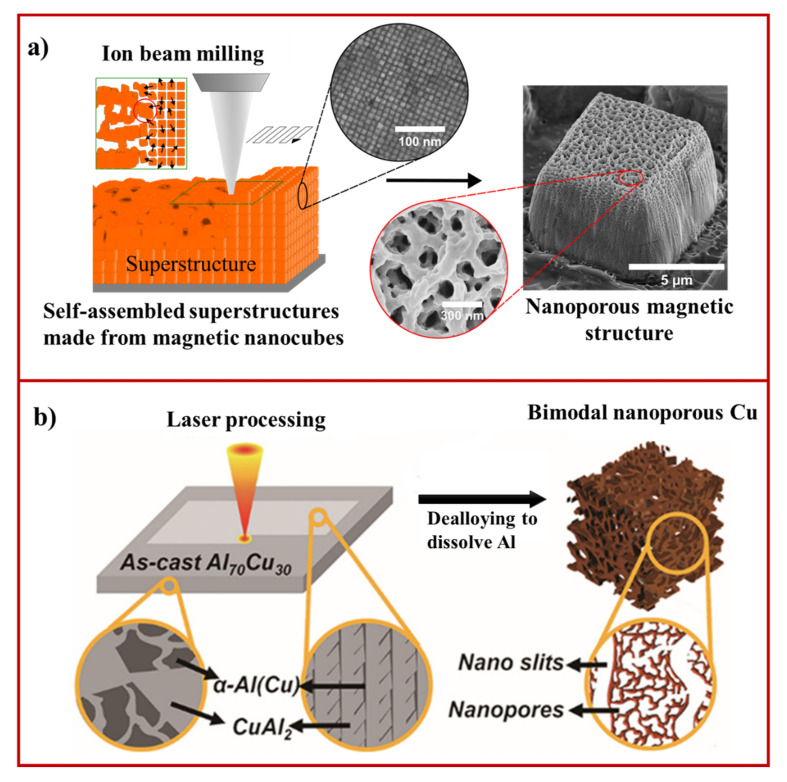
(**a**) The working principle of nanoporous fabrication using focused ion beam milling and melting process (adapted from [127], Copyright 2018 Royal Society of Chemistry); (**b**) schematic illustration displaying the fabrication of bimodal nanoporous Cu by a combined strategy involving laser processing and chemical dealloying (adapted from [129], Copyright 2022, Elsevier).

Recently, we established a novel strategy to fabricate nanoporous materials using the focused ion beam method (Figure 5a) [127]. Instead of bulk substrates, predesigned self-assembled magnetic superstructures made from cobalt ferrite nanocubes via self-assembly at air–liquid interface were used [128]. When a focused ion beam of voltage 30 kV was exposed to self-assembled magnetic superstructures, the porosity evolved in the self-assembled superstructures because of the combined effect of milling and melting (Figure 5a). The pore size (10–1 nm) was found to increase linearly with increasing ion beam voltage and ion doses. In addition, the distribution of pore size was also tuned by changing the shape of nanoparticles from nanocubes to spherical nanoparticles. This approach can be termed as generic for fabricating nanoporous materials from nanoparticles of different materials and shapes without any need for further dealloying. The scalability of this process can be achieved by replacing focused ion beam with broad ion beam technology.

## 7. Laser-Induced Fabrication of Nanoporous Materials

Laser techniques have widely been employed to generate micro- and nanopatterned surfaces [130], microfluidic channels [131], and tiny photonic waveguide structures [131]. Laser irradiation in combined with dealloying strategies have been explored for fabricating nanoporous materials. In the work of Gu et al., the fabrication of nanoporous manganese was demonstrated by laser cladding, followed by selective electrochemical dealloying [132]. In this work, Mn-Cu precursor alloy of thickness 1 mm was prepared by mixing Mn and Cu elemental powder and depositing it on the mild steel substrate. Laser power of 1.2 kW and scanning speed of 6 mm/s were used to achieve low diffusion cladding. The electrochemical dealloying of the laser processed specimen led to the formation of nanoporous MnCu. The pore size and backbone size of porous structures could be tuned by changing the electrochemical dealloying time from 30 min to 150 min. Additionally, strategies such as chemical oxidation and femtosecond laser ablation have been demonstrated to fabricate nanoporous anatase TiO_2_ on a microstructured Ti-based substrate [133]. Firstly, a femtosecond laser was used to generate a primary microarray patterned Ti surface. In the next step, nanoporous TiO_2_ was produced by the chemical oxidation (H_2_O_2_) of femtosecond laser-treated Ti surface. Finally, the nanoporous surface was thermally annealed to form anatase TiO_2_ without significant change in the morphology. The annealed anatase TiO_2_ showed improved photocatalytic activity and excellent stability under ultraviolet-visible (UV-Vis) light irradiation. Recently, a combined strategy including laser processing and dealloying has been developed to fabricate homogenously bimodal nanoporous Cu (Figure 5b) [128]. This work achieved laser processing of Al_70_Cu_30_ alloy by scanning the surface with fiber laser power of 6 kW (beam diameter ~0.5 mm). Later, the laser processed surface was chemically dealloyed in HCl solution (1 wt.%) to selectively remove Al, resulting in bimodal nanoporous Cu formation. The findings from this study revealed the critical role of laser-induced microstructural modification in the production of bimodal nanoporous structures. It should be noted that laser does not contribute to the porosity evolution in exposed materials. The laser processing technique has primarily been used for creating micro-and nanostructured surfaces with composition control. The nanoporosity in laser-processed samples evolves by chemical or electrochemical dealloying processes. Therefore, laser-induced nanoporous material fabrication requires additional techniques for creating nanoporosity in the materials, thus, limiting their applicability to produce nanoporous materials from a wide range of functional metals and metal oxides.

## 8. Biomedical Applications of Nanoporous Materials

Nanoporous materials have several biological and medical applications including biosensing, delivery of biological molecules, antimicrobial properties, dialysis, developing novel medical devices for orthopedics, and neural implants [134]. It has been shown that nanostructured materials can facilitate and increase cell growth, tissue remodeling, angiogenesis, and antimicrobial properties. Xu et al. demonstrated that Ti-based alloys modified by nanoporous oxidation to form nanopores and nanotubes on the substrates could facilitate enhanced cell adhesion, proliferation, and matrix deposition as compared with unmodified substrates, providing favorable conditions for tissue growth and osseointegration [135]. Furthermore, He et al. developed nanoporous surfaces by anodizing of Ti surface to generate the titania nanotubular structure to investigate the effects of nanotopography on osteogenesis. It was shown that the nanotopographical features promoted macrophage recruitments around the implants and inhibited osteoclast activity. The nanoporous Ti implants facilitated the secretion of cytokines that promoted mineralization, inhibited the activity of osteoclasts through the integrin signaling pathway, and regulated improved implant osseointegration [136]. 

Nanoporous materials are also being explored for the applications of neural prosthesis and bioelectronic medicine. Neural electrodes are considered to be the key communication bridge to detect and control neural activity between the body and an external device. It has been shown the electrodes with nanoporous substrates have the ability to reduce impedance and increase conductivity as compared with the smoother electrodes. A similar study reported by Shuang et al. demonstrated that Tungsten electrodes with nanoporous features showed reduced signal attenuations indicating enhanced consistency as compared with the smother electrodes [137]. These studies further suggests that the nanoporous materials have greater applications in bioelectronics space, since they have the potential to exhibit excellent electrochemical properties, biocompatibility, and stability [138].

Apart from facilitating biocompatibility, these materials have also been shown to inhibit infections without using antibiotics which is crucial and beneficial for the design and development of medical implants. Studies by Ge et al. have shown that fabrication of nanoporous substrates with nanopillar arrays could inhibit the adhesion, growth, and proliferation of microbes such as *S. aureus* and *E. coli*. The study attributed the antibacterial effect to the spatial confinement size effect and the availability of limited contact area, thus, hindering the microbial activity [139]. Nanoporous materials are also the center of attraction for drug and biomolecule delivery. Some of the examples investigated include nanoporous anodic alumina, porous silicon, and titanium. These materials have shown to act as excellent reservoirs for drugs and biomolecules due to their ability to minimize loss, biocompatibility, and their inherent physical and chemical properties [9]. Similarly, Bae et al. showed that stainless steel fabricated with nanoporous surface for orthopedic implants and stents demonstrated enhanced capabilities to store drugs and maximize its efficacy [140]. Furthermore, it has been shown that the nanoporous materials possess high surface area, tunable pore sizes, and have the capabilities to demonstrate controlled release of drugs and pesticides enabling these materials to be used in the pharmaceutical and agriculture industries [141]. 

## 9. Conclusions and Outlook

In this review, we have summarized recent developments and various fabrication strategies explored towards the fabrication of nanoporous materials from metals, metal oxides, and polymers. The dealloying approach has been extensively investigated to produce nanoporous metals and metal oxides with controlled pore sizes, porous structures, and chemical composition. Although dealloying is very simple and yields mechanically robust nanoporous materials, the approach cannot be considered to be a generic technology to fabricate a wide range of materials, including soft materials. Moreover, the use of toxic chemicals used during fabrication, careful preparation of alloy, and limitations with industrial scalability restrict applying the technique to a range of applications. Templating methods can be considered to be an alternative method to dealloying approaches because of their capability to produce nanoporous inorganic, polymers, or composite materials. However, the mechanical stability of nanoporous structures is compromised and hinders their broader practical applications, since the removal of template can destabilize the nanoporous structures. 

Nanoporous materials truly encompass an ever-expanding list of applications, ranging from agriculture to biosensing, tissue engineering, biomedical implants, energy, and environmental applications. Advanced fabrication technologies have allowed for growth in these areas due to their tailorability, highlighting the fact that different applications require different sets of criteria out of nanoporous materials (i.e., pore size, pore structure, crystallinity, and ordered vs. disordered structures). For example, ordered nanoporous materials prove much more efficient for optoelectronic applications because the interaction of electromagnetic waves with ordered nanopore structures can be controlled by the periodicity of nanopores. Ordered nanoporous materials are desirable for membrane and separation applications over disordered nanoporous materials. However, the disordered nanoporous materials are mechanically robust as compared with ordered nanoporous materials and, thus, can be used for the applications requiring excellent mechanical stability. Microporous materials are suitable for catalysis, sensing, and drug delivery applications. However, mesoporous materials can be used as adsorbents to remove pollutants from water and storages of gases. When nanoporous materials are exposed to harsh environments, their physical and chemical properties can degrade over time. To protect these nanoporous from degradation or aging, a coating of stable metal or doping of metal into the nanoporous structures is required to enhance the resistance towards aging.

In the last decade or so, significant progress has also been made in developing new strategies for producing nanoporous materials, including additive manufacturing, ion beam, and laser processing. Among these approaches, additive manufacturing approaches hold great potential to be employed for fabricating nanoporous materials of complex architecture with excellent mechanical stability. This is because the manufacturing industries have already adapted additive manufacturing technology. Additive manufacturing also enables us to develop digitally controlled next-generation nanoporous materials with desirable pore size, as well as material, architecture, and physical properties suitable for particular applications. Further work is necessary to maximize the potential benefits offered by additive manufacturing. In particular, the challenges associated with generic and environmentally friendly fabrication technology (capability to produce nanoporous materials independent of material choice without the use of toxic chemicals and materials recycling) and industrial-scale manufacturing are yet to be addressed. To minimize the environmental impact, new nanoporous manufacturing technologies should be designed and optimized to reduce the reliance on toxic and harsh solvents. Therefore, we expect that the integration of additive manufacturing with microwave-based and ion beam-induced fabrication technologies will enable the development of generic and scalable digital-assisted technologies with minimal environmental impact and foster the synthesis of nanoporous materials from recycled materials. 

## Figures and Tables

**Table 1:** List of fabrication techniques for nanoporous materials.

Fabrication Technique		Advantages	Disadvantages
Dealloying	Chemical	- Simple and no requirement of complex instrumentation - Control over the size of pores and ligament - Fabrication of metal and metal oxide nanoporous materials - Low temperature	- Coarsening (i.e., reduced surface energy/area and degradation of physical properties - Difficulty in fabricating non-metallic materials - Use of corrosive solvents - Impurities from less noble metal - Time consuming and no control over the composition of nanoporous structure - Not suitable for materials of similar chemical reactivity
	Electrochemical	- Better control over porosity and chemical composition than chemical dealloying - Low temperature	- Time consuming, harsh corrosive solvents, and difficult scalability - Limited to thin film - Not suitable for materials of similar electrochemical potentials
	Liquid Metal	- More efficient than chemical or electrochemical dealloying and more environmentally friendly - Faster due to operation at higher temperatures	- Requires high temperatures - Unavoidable thermal coarsening - Inapplicable for materials with similar solubilities
	Vapor Phase	- Facile and environmental fabrication technique for synthesizing nanoporous materials - Recycling metal from precursor alloy - No chemical waste	- Vacuum chamber limits industrial scalability and high-throughput production - High temperature process - Inapplicable for materials with similar vapor pressure
Templating	Soft	- Good control over geometry, pore size, and architecture - Simple methodology	- Collapse of nanoporous structures after the removal of soft template - Low mechanical stability - Low thermal stability of soft template - Not scalable approach - Low yield
	Hard	- Stable nanoporous structures after the removal of hard template - Retaining hard geometries	- Less tunability in the size and structures of nanopores - Low yield - High cost and require multiple steps - Not scalable - Low mechanical stability of nanoporous materials
Microwave-Based Fabrication		- High yield, purity, and selectivity - Nanoporous fabrication from metal, metal oxide, polymer and metal organic framework - Tunability in the size of nanopores and backbone structure via time and microwave power - Rapid process	- Low yield of nanoporous materials - Low depth penetration of microwave irradiation limiting the industrial scale production
Additive Manufacturing		- Tunable architecture - Ability to produce materials over multiple lengths - High level of reproducibility and industrial scalability - High mechanical robustness - Ability to create compositionally gradient hierarchical porous materials	- Trade-off between mechanical stability and porosity/pore size - Requirement of chemical dealloying or other fabrication strategy to achieve nanoporosity limits their industrial scale production
Ion Beam-Induced Fabrication		- Fine control over pore size distribution, and interconnected pore network - No toxic chemicals - Independent of material choice - Requires no additional steps - High mechanical stability - Rapid process	- Contamination of backbone elements - Scalability - Requires a vacuum system
Laser-Induced Fabrication		- No need for vacuum - Rapid laser processing of bulk materials - Generates micro- and nanostructures with tunable sizes and morphologies	- Require additional step of dealloying process to generate porosity in the materials - Limited to selective materials - Scalability - Use of hard chemicals in dealloying step

## Data Availability

Not applicable.
